# Prospective association between self-reported life satisfaction and mortality: Results from the MONICA/KORA Augsburg S3 survey cohort study

**DOI:** 10.1186/1471-2458-11-579

**Published:** 2011-07-20

**Authors:** Maria E Lacruz, Rebecca T Emeny, Jens Baumert, Karl H Ladwig

**Affiliations:** 1 Helmholtz Zentrum München, German Research Center for Environmental Health, Institute of Epidemiology II, Neuherberg, Germany; 2 Department of Psychosomatic Medicine and Psychotherapy, Klinikum rechts der Isar, Technische Universität, Munich, Germany

## Abstract

**Background:**

To identify factors which determine high life satisfaction (LS) and to analyse the prognostic influence of LS on mortality.

**Methods:**

Data collection was conducted on 2,675 participants, age 25-74 years, as part of the MONICA Augsburg Project 1994-95. Multivariate logistic regression analyses were used to determine factors associated with high LS (measured with one item, 6-level Likert scale, where "high" = very satisfied/most of the time very satisfied with ones personal life). After 12 years mean follow-up, a total of 245 deaths occurred. We calculated age- and sex-adjusted incident mortality rates per 10,000. Hazard ratios (HRs) were estimated from Cox proportional hazards models.

**Results:**

Independent determinants of LS were income, health-perception, and social support, as well as somatisation, anger or depressive symptoms (all p < 0.05). Participants with higher LS (n = 721, 27%) benefited the most with respect to absolute mortality risk reduction (higher LS = 67; mid = 98; low = 140 per 10,000). The sex-stratified analyses indicated an independent association of higher LS and survival for men (HR 0.55; 95% CI 0.37 - 0.81) but not for women.

**Conclusions:**

Baseline assessment demonstrated that psychological, social and life-style factors, but not somatic co-morbidities, were relevant determinants of LS. Moreover, the analysis showed that men with higher LS have a substantial long-term survival benefit. The observed association between LS and mortality may be attributed to common underlying causes such as social network integration and/or self-rated health.

## Background

Much research has been done on the prospective associations between negative affective states, physical health, and total mortality [1-3]. In contrast, there has been little research linking well-being with physical health, although limited evidence points to the association of well-being with greater health and longevity [4-6]. It is generally accepted that there are three independent facets of positive well-being: positive affect, negative affect and life satisfaction (LS) [7]. LS measures vary in their composition, but generally, they identify trait levels of positive affect as well as cognitive assessments of the extent to which a person's life matches his or her expectations [8]. Although there have been studies examining the connections between overall well-being and health, we focus on life satisfaction because it reflects subjective perceptions of success and happiness [8] and thus may be more stable than measures of positive affect [9].

LS has been shown to be associated with lower morbidity and mortality among older community-dwelling individuals [5]. Furthermore, a robust negative association of LS with morbidity in both healthy and ill populations has been demonstrated [6]. In addition, LS seems to protect individuals against physical decline in old age [10]. While there is an increased interest in the study of LS and the health consequences of positive functioning, to our knowledge, no study to date has specifically examined sex-specific aspects of LS in a population-based sample with a broad age span (25-74 years of age) and a long follow-up.

Therefore, we aimed to identify characteristics that are associated with an individual's LS in a German population, as well as to determine the effect of LS on mortality. The present study utilizes a broad range of parameters based on the MONICA/KORA cohort study to elucidate socioeconomic, psychological and health-related determinants of LS. Furthermore, we assessed the absolute and relative mortality risk of LS over a mean follow-up time of 12 years.

## Methods

### Study design and population sample

The data were derived from the population-based MONICA (Monitoring Trends and Determinants on Cardiovascular Diseases Augsburg) S3 survey conducted in 1994-95 [11]. The MONICA Augsburg survey was part of the multinational WHO MONICA project [12]. The study area is located in southern Germany and comprises the city of Augsburg and two surrounding counties, with approximately 600,000 inhabitants, in a mixed urban and rural area. Written informed consent was obtained from each study participant, and the study was approved by the local ethics committee. For this survey, a sex and age-stratified, random, representative sample of 6,481 eligible subjects was drawn from the population, of which a total of 4,856 individuals aged 25 to 74 years were enrolled in the study (response rate: 74.9%).

A total of 2,698 participants completed the psychological questionnaire. Among those, 23 participants who had missing values on at least one of the covariates were excluded. Therefore, the study population of the present analysis included 2,675 participants (1,423 men and 1,252 women) aged 25 to 74 years. A drop-out analysis revealed that subjects who refused to answer the questionnaire were more often women (p < .005) and were generally older (p < .001) than those who were included in this study.

### Index population

LS was measured by asking the following question: "How satisfied were you with your personal life in the last month?" A similar one-item measure of subjective well-being is thoroughly validated and widely used in German [13]; Canadian [14] and Jamaican [15] surveys. Answer categories for the LS item were: very satisfied ( = 5); most of the time very satisfied ( = 4); usually satisfied ( = 3); partially satisfied ( = 2); usually unsatisfied ( = 1); very unsatisfied ( = 0). Based on the skewed distribution of the sample, we created a variable with three LS categories: high (*very satisfied *and *most of the time very satisfied*), medium (*usually satisfied*) and low (*partially satisfied*; *usually unsatisfied*; *very unsatisfied*), which roughly followed the tertiles of the distribution.

### Covariates

#### Socio-demographic

These variables were determined in the standardised interview. Equivalent household income was calculated as [*total household income ÷ (household size)^0.36^*] [16].

#### Risk factors for cardiovascular diseases (CVD)

A nonfasting, venous blood sample was collected from all participants in resting position. Total serum cholesterol and high-density lipoprotein cholesterol were analysed by enzymatic methods (CHOD-PAP; Boehringer Mannheim, Germany).

Diabetes mellitus was defined if glucose concentrations were ≥ 11.1 mmol/l, or glycated haemoglobin (HbA1c) > 7%, or use of anti-diabetic medication was confirmed. Actual hypertension was defined as blood pressure values ≥ 140/90 mm Hg, or use of antihypertensive medication.

#### Lifestyle and co-morbidities

A *physical activity restriction *was considered when someone felt that their physical activity was limited due to a health problem. The "healthy nutrition" score is based on a food frequency questionnaire, from which a score of 0 to 30 is calculated [17]. Presence of self reported illness was determined in the interview.

#### Psychological variables

Twenty-four somatic complaints were measured with the "von Zerssen symptom check list" [18]. Depressive symptomatology, measured with the DEEX-scale was assessed using a subscale from the von Zerssen affective symptom check list [19]. Subjects in the top tertile of the depressive symptom distribution (n = 982 vs. n = 1693) were considered as an index group for subjects with depressed mood [19].

Perceived health was assessed in the interview with seven questions that provided information about the following domains: self-rated health, health-status, a judgement of health status compared to others, vulnerability healthwise, responsibility for own health, contact last month to a mental health provider, tension, and time pressure. Anger was evaluated with a modified version of the STAXI questionnaire, sub-scores for disposition to irritation, anger expression - out, anger expression - in, and anger control were calculated [20]. Type-A personality was assessed using the Framingham Type-A scale [21]. Social support was characterised with the Berkman-Syme's Social Network Index [22]. The components of the index are weighted in an algorithm resulting in four categories as suggested previously; the categories were further condensed to form a dichotomous variable: low vs. high social support.

### Study endpoints and follow up

Vital status was assessed for all sampled persons in a follow-up study in 2008. By December 31, 2007, 245 persons (183 men, 62 women) had died. The study population was followed for an average of 12 years (S.D. 2.1). Death certificates were obtained from local health departments and coded for the underlying cause of death by a single trained person, using the 9th revision of the International Classification of Diseases (ICD-9) [23].

### Statistical methods

#### Descriptive analysis and determinants of LS

The χ^2 ^test was used to examine associations between categorical variables. To evaluate the association of all previously mentioned factors with LS, logistic regression models were calculated controlling for age and sex. To reduce confounding that may arise from correlated variables, and also to reduce the ratio of variables to data, we excluded variables that were strongly correlated with each other (Spearman's r > .7) and those variables which were not significantly different among the participants of each of the three LS categories (χ^2 ^test with Bonferroni correction for 38 test, p < 0.001). A stepwise variable selection with backward elimination (entry criterion p < 0.25 in the univariate model and stay criterion p < 0.05 in the end model) was performed for "high LS" versus medium/low. We assessed the validity of our classification of LS on the basis of statistically significant determinants by measuring the area under the corresponding receiver operating characteristic (ROC) curve (AUC or c statistic). Additionally, to allow for comparisons across dependent variables and were interpreted according to Cohen's effect size index, with 0.2 indicating a small difference, 0.5 a moderate difference, and 0.8 or more a large difference [24,25].

#### Absolute mortality risk

We calculated age- and sex-adjusted incident mortality rates per 10,000 on the basis of 3 age groups (25-39, 40-59 and 60-74 years). Age standardisation was carried out, using the direct standardisation method. The standard population to which the age distribution of sub-groups was adjusted was the entire survey population. The Cochran-Armitage exact test for trend was used to determine if there was a different trend for mortality on each LS subgroup.

#### Relative mortality risk

Hazard ratios (HRs) comparing the middle and lower LS tertiles with the upper LS tertile are reported together with their 95% confidence intervals (CIs). Different models were built up to check for the effect of LS on mortality: a) crude model considering sex, age and LS; b) cardiovascular model considering the crude model and cardiovascular risk factors (alcohol consumption; obesity; hypertension; smoking; physical activity and hypercholesterolemia); c) health model considering the crude model and health variables (presence of comorbidities and use of medication); d) psychological considered crude model and psychological determinants of LS (presence of somatic symptoms, depressed mood, impaired self-rated health, impaired health status, disposition to irritation, anger and low social network index) and e) social considered crude model and social determinants of LS (low net income). Additionally, a sensitivity analysis was run with participants not suffering at baseline from cardiovascular diseases (angina pectoris, myocardial infarction or stroke, n = 89). Analyses were run for all participants and sex-stratified. All variables were categorical and met the proportional hazards assumption. In the Cox analysis, the follow-up time from enrolment in the study to the event (for cases) or to the last contact for outcome information (for non-cases) was modelled. Non-cases were censored at the end of their follow-up time. We assessed the relative goodness of fit of our Cox models by Akaike information criterion (AIC).

Significance tests were two-tailed and unless otherwise stated *p *values <.05 are statistically significant. Data were analysed using SAS 9.2 software (SAS Institute Inc., Cary, NC).

## Results

### Gender and age groups comparisons

**Figure 1:**
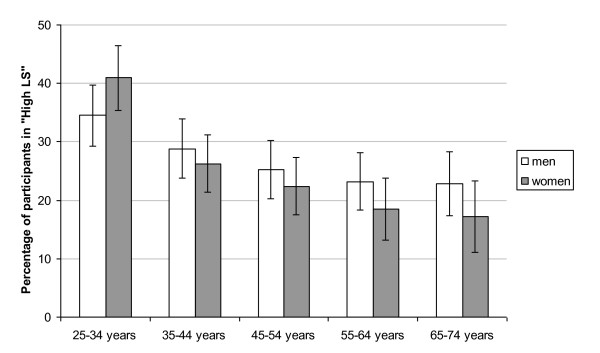
**Percentage (95%CI) of participants in the High LS group, by gender and age group**.

The 2,675 participants had a mean age of 47 years (SD 13.6) and 53% were male. A total of 721 participants (27%) were classified in the "high LS" group which consisted of 54% men with a mean age of 44 years (SD 13.5). A total of 1,485 participants (55%) were classified in the medium category including 56% male with a mean age of 49 years (SD 13.3). Finally, 469 participants (18%) were classified in the "low LS" group which included 44% male with a mean age of 44 years (SD 13.2). No sex differences were found in percentage of high LS, either in total or age-stratified groups, as can be seen in Figure 1. With increasing age, the percentage of participants in the high LS subgroup declined for all participants (χ^2 ^= 45.88, p < .0001) as well as for men (χ^2 ^= 12.3, p = .0004) and women (χ^2 ^= 39.8, p < .0001).

### Differences in socio-demographic, somatic and psychological factors between subgroups of LS

Additional File 1: table S1 shows the differences in CVD risk factors, life-style and co-morbidities, socio-demographic variables and psychological factors between high, medium and low subgroups of LS. Severe chronic disease conditions (diabetes, myocardial infarction, stroke or cancer) were not significantly different between the index population of high LS (N = 721) and the other LS subgroups. On the contrary, most psychological variables, e.g. depressive symptomatology, health perception, or anger, showed significantly different prevalences between subgroups.

### Determinants of LS

The results of the multivariate logistic regression analyses (Table 1) indicate that independent determinants of higher LS included increased net income (p=.001), good self-rated health (p=.001), good health status (p=.01), and social support (p < .001) along with low levels of somatic complaints (p=.001), and not having depressive symptomatology (p < .001), a disposition to irritation (p < .01), or anger expression - in (p=.02). The area under the ROC curve for the screening based on these 8 domains was 0.73, which suggests that the combination of these 8 variables correctly identifies in 73% of the cases, the subjects belonging to the high versus the combined medium and low subgroups.

**Table 1:** Logistic regression [OR (95%CI)] between socio-demographic variables, psychological variables and LS, adjusted for age and gender (N = 2675).

	High (N = 721) vs. medium and low (N = 1954)	Cohen's effect size index (High vs. medium/low LS)
**Socio-demographic**		

Low net income	0.73 (0.60 - 0.88)	0.19

**Psychological variables**		

Somatic complaints	0.68 (0.54 - 0.86)	0.51
Depressive symptomatology	0.36 (0.28 - 0.46)	0.69
Impaired self-rated health	0.56 (0.39 - 0.79)	0.46
Impaired health-status	0.50 (0.29 - 0.86)	0.44
Disposition to irritation	0.78 (0.65 - 0.94)	0.30
Anger expression - in	0.79 (0.65 - 0.96)	0.21
Low social network index	0.59 (0.49 - 0.70)	0.35
**C**	**.73**	

Only significant variables for each group are shown.

### Mortality

After an average follow-up period of 12 years (S.D. 2.1), a total of 245 participants in the study population of 2,675 participants had died among them 183 men and 62 women.

#### Absolute risk of LS on Mortality

**Figure 2:**
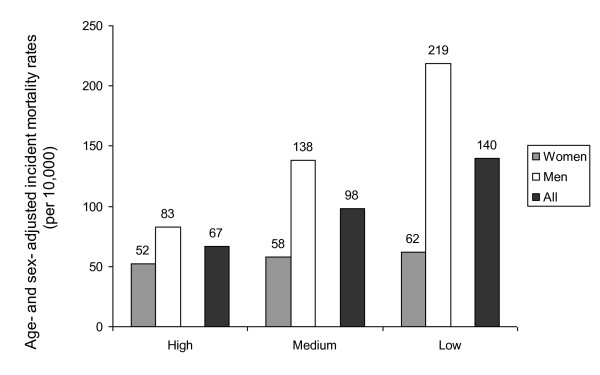
**Absolute mortality risks for each LS tertile by gender and for the entire sample**.

In the full sample, age- and sex- adjusted incident mortality rates (per 10,000) increased in a stepwise fashion with decreasing levels of LS; from 67 in the high subgroup, to 98 in the medium subgroup, to 140 in the low subgroup (p for trend =.01) (Figure 2). However, the sex-adjusted analysis revealed that the survival benefit of LS was only due to the male participants (219 in low LS, to 138 in medium LS, to 83 in high LS) (p for trend=.0006). For women, no effect of LS on mortality could be observed (p for trend = 0.3).

#### Relative risk of LS on Mortality

As shown in Table 2, significant relative risk reductions for all-cause mortality can be reported for higher LS (relative risks for all adjusting variables are shown in additional File 2 : table S2, table S3 and table S4). For all participants, the HR was 0.61 (95% CI 0.44 - 0.86) in the crude Cox analysis model (adjusted for age and sex) indicating a 39% survival benefit for participants in the higher LS subgroup. Stratification by sex resulted in an independent association of higher LS and survival for men (HR 0.55, 95% CI 0.37-0.89) but not for women.

**Table 2:** Predictors of all-cause mortality in participants with high vs medium/low life satisfaction.

	No. of subjects/No. of deaths	Crude model^a^	Model cardio-vascular risk factors^b^	Model health^c^	Model psychological determinants LS^d^	Model social determinants LS^e^
**All-cause mortality**						

All	2675/245	0.61 (0.44 - 0.86) **	0.68 (0.49 - 0.95) *	0.64 (0.46 - 0.90) **	0.77 (0.54 - 1.09)	0.63 (0.45 - 0.88) **
AIC		3513.3	3471.7	3466.1	3489	3506.7

Women	1252/62	0.93 (0.49 - 1.76)	0.95 (0.50 - 1.80)	0.88 (0.47 - 1.65)	1.13 (0.58 - 2.17)	0.93 (0.50 - 1.76)
AIC		772.4	776.7	764.9	772.4	773.9

Men	1423/183	0.55 (0.37 - 0.81) **	0.61 (0.41 - 0.91) *	0.58 (0.39 - 0.86) **	0.68 (0.45 - 1.02)	0.57 (0.38 - 0.84) **
AIC		2456.5	2421.5	2415.4	2442.5	2449.2

**All-cause mortality for participants with no baseline diagnosis of cardiovascular diseases (angina pectoris, myocardial infarction or stroke; n = 89)**						

All	2586/156	0.62 (0.41 - 0.94) *	0.71 (0.47 - 1.07)	-	0.80 (0.52 - 1.22)	0.64 (0.42 - 0.96) *
AIC		2816.9	2819.2		2847.9	2856

Women	1229/39	1.12 (0.53 - 2.38)	1.23 (0.58 - 2.63)	-	1.24 (0.57 - 2.71)	1.13 (0.53 - 2.38)
AIC		659	661.9		660.5	660.7

Men	1357/117	0.52 (0.32 - 0.85) **	0.60 (0.37 - 0.99) *	-	0.68 (0.41 - 1.13)	0.54 (0.33 - 0.88) *
AIC		1964	1929.8		1960	1957

Hazard Ratios [HR (95%CI)] for life satisfaction for all-cause mortality. Data are separated for all participants and only healthy participants.

* p < .05; ** p < .01

^a ^adjusted for sex (only for all participants) and age

^b ^adjusted for sex (only for all participants), age, alcohol consumption; obesity; hypertension; smoking; physical activity and hypercholesterolemia

^c ^adjusted for sex (only for all participants), age, presence of comorbidities and use of medication

^d ^adjusted for sex (only for all participants), age, presence of somatic symptoms, depressed mood, impaired self-rated health, impaired health status, disposition to irritation, anger and low social network index

^e ^adjusted for sex (only for all participants), age and low net income.

Table 2 demonstrates a unique effect of a model of psychological determinants on the relationship between LS and mortality compared to models adjusted for cardiovascular risk factors, health factors and social factors. For men, LS showed an effect on mortality in models adjusted for cardiovascular risk factors, health factors and social factors, with a similar strength of association in all models (HRs from 0.57 to 0.61). The fact that even after adjustment for cardiovascular risk factors or health factors, the relationship between mortality and LS remained significant (HR 0.61; 0.41 - 0.91 and HR 0.58; 0.39 - 0.86 respectively), suggests that neither CVD risk factors nor health factors are confounders in the association between LS and mortality. Controlling for psychological factors affected the relationship between LS and mortality as the strength, significance and, in the case of women, the direction of the effect of LS on mortality was changed when psychological factors were included in the model. This pattern suggests a confounding role of psychological variables in the association between LS and mortality.

#### Sensitivity analysis of concurrent illness with LS

We repeated the mortality analysis excluding subjects with pre-existing cardiovascular disease (N = 89), leaving a total population of N = 2,586, among whom 156 died in the follow-up period.

Absolute risk of LS on mortality in a disease-free population: In the full sample, age- and sex-adjusted incident mortality rates (per 10,000) increased from 47 in the high LS subgroup to 63 in the medium LS subgroup to 102 in the low LS subgroup (p for trend = 0.09). This trend became significant for men only (53 in high LS to 90 in medium LS to 167 in low LS; p for trend = .005) and lost significance in women (p = .28).

Relative risk of LS on mortality in a disease-free population (Table 2): Essentially identical results were observed in models using only healthy participants. Significant risk reductions for all-cause mortality can be reported for higher LS for men but not for women. In the crude model, the HR for men was 0.52 (0.32 - 0.85). No significant associations between LS and mortality were found in the psychological model.

## Discussion

### Survival benefit

The major finding of this study was that participants with higher LS benefited the most with respect to absolute mortality risk reduction (higher LS = 67; mid = 98; low = 140 per 10,000). Furthermore, higher LS was independently associated with survival in men (HR 0.55, 0.37-0.81) but not in women. The present analysis demonstrates that the relationship between LS in men and mortality persists even after adjustment for baseline risk factors and severe sustained co-morbidities, which likely provides a conservative estimate of the overall effect of LS on survival. These findings suggest that for men regardless of their somatic and psychological health, being satisfied with one's life is protective against mortality. This is in agreement with previous reports, which clearly show the association between a global subjective perception of one's own health and mortality. These studies also found a significant, independent association that persists even after adjustment for health status indicators and other relevant covariates [26]

The finding that LS was not associated with mortality in women is interesting. Although there were similar frequencies of high LS in both sexes in our study sample, there was a clear association of LS and mortality for men but not for women. Only few publications have addressed sex differences and found a similar sex-specific effect [27,28]. It has been suggested that in men, morbidity-related factors are the most important predictors of mortality while in women the predictors were spread over more domains [29]. Additionally, Koivumma-Honkanen *et al*. have speculated that females may be more capable of coping with psychological distress than males, thus avoiding fatal consequences [28]. Furthermore, the reasons for these differences may also include different lifestyles and different biological vulnerability [29]. Still, in considering mortality, statistical power may be compromised by the small number of deaths in women (62 deaths out of 1,252 participants). Nevertheless, this difference deserves further investigation.

Inclusion of individuals with pre-existing illness is potentially problematic because their perspective on life may likely be negatively affected by their disease experience, and thereby could drive down LS in the entire population. Additionally pre-existing illness is likely to be associated with both LS and mortality. Therefore, a sensitivity analysis was performed with exclusions made for patients suffering from cardiovascular disease at baseline (n = 89) and, against expectations, returned essentially identical results. Healthy participants in the high LS tertile showed a 38% mortality risk reduction compared with those in the lower tertile. These values are within the range reported previously in a meta-analysis: mean HR of 0.82 (CI = 0.76-0.89) of 21 studies with healthy populations [6]. Additionally, it has been shown that LS significantly predicted a lowered risk of all-cause and natural cause mortality, and this association is especially salient in the healthy subsample [30]. Again, as observed in the entire cohort, in sex-stratified analyses this association remained true for men, but not for women.

Our data suggest, as previously reported, [6] that LS has a favourable effect on survival in healthy and disease populations, which was lost after adjusting for other psychological determinants. The fact that LS lost significance in the psychological model, could have been caused in part by the well-established association between self-rated health and mortality [26], that may have weakened the relation between LS and mortality. Indeed, only when either self-rated health or social network index were excluded from the psychological model, was the LS association with mortality restored (data not shown). The exclusion of none of the other variables from the "psychological model" (including depressed mood) did not modified the association between LS and mortality.

### Determinants of LS

The present study provides a broad range of somatic and psycho-social determinants to elucidate both the determinants of LS as well as possible underlying factors that may explain the substantial survival benefit of LS. Consistent with previous reports [31], a significant decline in LS was observed across the life span of women in our population. However, the lack of decline in LS among men reported in the literature [32] was not seen in our population. How LS changes with age is an intriguing question, especially in light of prior findings that it improves from middle age onward, even in the face of physical health decline; little is known about the determinants of this pattern [33,34]. The decline in LS across life span for men and women could be partially explained by the fact that older people are more often ill and health-related factors play an important role in LS. Indeed, when the analyses are repeated only for "healthy participants" (sensitivity analysis) we can see the previously reported U-shape pattern with lowest LS levels in middle 50s for both men and women [34] (data not shown).

It was previously shown that socio-demographic variables explain roughly about 8-15% of the variance in LS [35] and psychological and social characteristics explained 62% of the variance in LS [36]. The powerful impact of psychological and social characteristics as independent determinants of high LS is well illustrated in the present logistic regression analysis, where 73% of the variance in LS could be explained. An individual's positively-perceived health (lack of somatic complaints, good self-rated health, and good health status), a healthy psychological status (no depressed mood, anger disposition, or suppression of angry feelings) and good socio-demographic conditions (higher income, high social support) were associated with higher LS. Interestingly, although some co-morbidities (angina, insomnia, acute illness last week) were associated with differences in LS, none of these variables were relevant determinants of LS according to the logistic regression analysis.

The lack of association between co-morbidities and lifestyle factors (physical activity, diet) with LS in our comprehensive, holistic model may seem surprising. However, the relationship between well-being and medically-based health measures is still unclear. There is conflicting evidence with some studies showing that healthier people are more satisfied with life [37], and others which indicate that the relation between medically based health and well-being is weak [38]. Nonetheless, this lack of association between LS and ill health in our analyses may have been due to the small number of subjects with co-morbidities in our sample, which is a reflection of the population-based character of our sample.

### Strengths, limitations and guidelines for future studies

The present study has several important strengths. Foremost, it is a population-based sample, in which healthy and ill participants were included, well defined health outcomes, and inclusion of an exhaustive list of relevant covariates. The prospective study design allowed for a reasonable follow-up time to assess health outcomes. Some limitations, however, need to be addressed. In the present study, sub-syndromal depressive mood was assessed by the DEEX scale, which is a less rigorous instrument to assess depressed mood, although a recent re-examination of its validity and reliability is promising [19]. The assessment of LS with a one-item question is disputed, however, previous studies have used similar questions [13-15] and the factorial load in the total Satisfaction With Life Scale is very high (.82 to .89) [39]. The baseline measurement of life satisfaction assessed on average 12 years before follow-up provided strong risk estimates similar to other studies in which validated LS measurements in different time intervals were employed [6]. The inclusion rate for this study was 55% of all participants in the survey, potentially limiting the generalizability of our findings [40]. A cautionary note must be taken when interpreting prior findings on "positive" factors and health because it is still unclear from the literature whether "positive" traits are associated with better health or "negative" psychological traits are associated with worse health. The design of the current study, like any observational study that did not extend across the life course, cannot determine a causal relationship. However the results demonstrate an inverse association between LS and mortality.

## Conclusion

In summary, our cross-sectional analysis suggests that LS is essentially a subjective construct associated with social roles, psychological characteristics, and health perception, but not somatic factors. Moreover, in men LS has a substantial impact on long-term survival. Participants with higher LS benefited the most with respect to absolute mortality risk reduction (higher LS = 67; mid = 98; low = 140 per 10,000). Furthermore, higher LS was independently associated with survival in men (HR 0.55, 0.37-0.81) but not in women. The observed association between LS and mortality, wholly or in part, may be attributed to common underlying causes such as social network integration and/or self-rated health.

## List of Abbreviations

BMI: body mass index; CI: confidence interval; CVD: cardiovascular disease; DEEX: DEpression and EXhaustion subscale; HR: hazard ratio; ICD-9: international classification of diseases - 9^th ^revision; LS: life satisfaction; MONICA: Monitoring Trends and Determinants on Cardiovascular Diseases; KORA: Cooperative Research in the Region of Augsburg; OR: odds ratio; ROC: receiver operating characteristics; tot/HDL: ratio of total cholesterol to HDL cholesterol; WHR: waist hip ratio.

## Competing interests

The authors declare that they have no competing interests.

## Authors' contributions

MEL and KHL initially conceived the study and further developed the study objectives in collaboration with all co-authors. MEL wrote the initial draft of the manuscript and performed the analysis. JB and RTE were biostatistics advisor and made substantial contributions to the analytic approach. All authors were involved with drafting the final manuscript, and revising it as needed for important intellectual content. All authors read and approved the final manuscript.

## Pre-publication history

The pre-publication history for this paper can be accessed here:

http://www.biomedcentral.com/1471-2458/11/579/prepub

## Supplementary Material

Additional file 1 **Differences in socio-demographic, psychological and health variables between high, medium and low subgroups of LS**. Table S1. Differences in CVD risk factors, life-style and co-morbidities, socio-demographic variables and psychological factors between high, medium and low subgroups of LS [N (%)]. Click here for file

Additional file 2 **Multivariate Associations of LS with Mortality**. Table S2. Multivariate Associations [HR (95%CI)] of LS with Mortality for all-cause mortality (N = 2675). Table S3. Multivariate Associations [HR (95%CI)] of LS with Mortality for all-cause mortality in women (N = 1252). Table S4. Multivariate Associations [HR (95%CI)] of LS with Mortality for all-cause mortality in men (N = 1423). Click here for file
