# Supplementary material for: Prospective association between self-reported life satisfaction and mortality: Results from the MONICA/KORA Augsburg S3 survey cohort study
Source: BMC Public Health. 2011 Jul 20;11:579. doi: 10.1186/1471-2458-11-579 (PMC3155500; doi:10.1186/1471-2458-11-579)
Supplement: Additional file 1 — Differences in socio-demographic, psychological and health variables between high, medium and low subgroups of LS. Table S1. Differences in CVD risk factors, life-style and co-morbidities, socio-demographic variables and psychological factors between high, medium and low subgroups of LS [N (%)]. [file 1471-2458-11-579-S1.DOC]

Table S1. Differences in CVD risk factors, life-style and co-morbidities, socio-demographic variables and psychological factors between high, medium and low subgroups of LS [N (%)]. Factors with differences are marked in bold.

|  | **High**  **N=721** | **Medium**  **N=1485** | | **Low**  **N=469** | **p** | |
| --- | --- | --- | --- | --- | --- | --- |
| **Risk factors CVD** | | | | | | |
| Current regular smoker | 159 (22.05) | 340 (22.90) | | 140 (29.85) | .005 | |
| WHR #: ≥ 0.85 women; ≥ 1 men | 73 (10.12) | 223 (15.02) | | 69 (14.71) | <.01 | |
| Obesity: BMI * ≥ 30 | 124 (17.20) | 338 (22.76) | | 93 (19.83) | .12 | |
| Alcohol use: ≥ 20 g/d women;  ≥ 40 g/d men | 168 (23.30) | 322 (21.68) | | 91 (19.40) | .11 | |
| Diabetes mellitus | 18 (2.50) | 51 (3.43) | | 11 (2.35) | .93 | |
| Hypertension ≥ 140/90 mm Hg | 354 (49.10) | 882 (59.39) | | 208 (44.35) | .56 | |
| Total cholesterol ≥ 4.78 mmol/l | 585 (81.14) | 1297 (87.34) | | 385 (82.09) | .27 | |
| LDL-cholesterol ≥ 200 mg/dl | 510 (70.74) | 1149 (77.37) | | 336 (71.64) | .35 | |
| Tot / HDL ≥ 5.0 | 233 (32.32) | 594 (40.00) | | 139 (29.64) | .86 | |
| **Life style, co-morbidities** | | | | | | |
| **Physical inactive: <1 h/wk** | **315 (43.69)** | **806 (54.28)** | | **272 (58.00)** | **<.0001** | |
| **Physical activity restriction** | **122 (16.92)** | **400 (26.94)** | | **146 (31.13)** | **<.0001** | |
| Unhealthy diet: <14 | 230 (31.90) | 470 (31.65) | | 165 (35.18) | .30 | |
| Myocardial infarction | 7 (0.97) | 35 (2.36) | | 10 (2.13) | .10 | |
| Stroke | 7 (0.97) | 23 (1.55) | | 8 (1.71) | .26 | |
| Asthma | 14 (1.94) | 45 (3.03) | | 18 (3.84) | .05 | |
| Cancer | 8 (1.11) | 28 (1.89) | | 10 (2.13) | .15 | |
| **Angina pectoris** | **10 (1.39)** | **50 (3.37)** | **32 (6.82)** | | **<.0001** |  |
| **Insomnia** | **105 (14.56)** | **399 (26.87)** | **158 (33.69 )** | | **<.0001** |  |
| **Self-reported current acute illness** | **43 (5.96)** | **156 (10.51)** | **57(12.15)** | | **.0001** |  |

* BMI = weight (kg)/height^2^ (m)

# WHR = waist circumference (cm) / hip circumference (cm)

Table S1 cont.

|  | **High**  **N=721** | **Medium**  **N=1485** | **Low**  **N=469** | **p** | |  |
| --- | --- | --- | --- | --- | --- | --- |
| **Socio-demographic** | | | | | | |
| Females | 333 (46.19) | 655 (44.11) | 264 (56.29) | .004 | |  |
| Low net income (<1500 €) | 457 (63.38) | 1084 (73.00) | 329 (70.15) | .002 | |  |
| **Living alone** | **112 (15.53)** | **304 (20.47)** | **156 (33.26)** | **<.0001** | |  |
| Education less than 12 years | 421 (58.39) | 1014 (68.28.) | 310 (66.10) | .001 | |  |
| **Psychological variables** | | | | | |  |
| **High somatic complaints (≥ 7)** | **143 (19.83)** | **619 (41.68)** | **230 (49.04)** | **<.0001** |  | |
| **Depressive symptomatology** | **110 (15.26)** | **567 (38.18)** | **305 (65.03)** | **<.0001** |  | |
| **Impaired self-rated health** | **54 (7.49)** | **294 (19.80)** | **166 (35.39)** | **<.0001** |  | |
| **Impaired health-status** | **21 (2.91)** | **158 (10.64)** | **129 (27.51)** | **<.0001** |  | |
| **Worse health as others** | **35 (4.85)** | **123 (8.28)** | **100 (21.32)** | **<.0001** |  | |
| **Vulnerable healthwise** | **114 (15.81)** | **408 (27.47)** | **180 (38.38)** | **<.0001** |  | |
| **Irresponsible about health** | **228 (31.62)** | **562 (37.85)** | **209 (44.56)** | **<.0001** |  | |
| **Mental health care contact** | **8 (1.11)** | **10 (0.67)** | **23 (4.90)** | **<.0001** |  | |
| **Tension** | **85 (11.79)** | **272 (18.32)** | **212 (45.20)** | **<.0001** |  | |
| **Time pressure** | **214 (29.68)** | **465 (31.31)** | **231 (49.25)** | **<.0001** |  | |
| **Disposition to irritation** | **312 (43.27)** | **788 (53.06)** | **342 (72.92)** | **<.0001** |  | |
| **Anger expression - in** | **220 (30.51)** | **577 (38.86)** | **216 (46.06)** | **<.0001** |  | |
| Anger expression - out | 256 (35.51) | 528 (35.56) | 181 (38.59) | .33 |  | |
| No anger control | 192 (26.63) | 385 (25.93) | 148 (31.56) | .11 |  | |
| **Type-A personality** | **286 (39.67)** | **610 (41.08)** | **285 (60.77)** | **<.0001** |  | |
| **Low social network index** | **267 (37.03)** | **766 (51.58)** | **282 (60.13)** | **<.0001** |  | |
