# Supplementary material for: Prospective association between self-reported life satisfaction and mortality: Results from the MONICA/KORA Augsburg S3 survey cohort study
Source: BMC Public Health. 2011 Jul 20;11:579. doi: 10.1186/1471-2458-11-579 (PMC3155500; doi:10.1186/1471-2458-11-579)
Supplement: Additional file 2 — Multivariate Associations of LS with Mortality. Table S2. Multivariate Associations [HR (95%CI)] of LS with Mortality for all-cause mortality (N = 2675). Table S3. Multivariate Associations [HR (95%CI)] of LS with Mortality for all-cause mortality in women (N = 1252). Table S4. Multivariate Associations [HR (95%CI)] of LS with Mortality for all-cause mortality in men (N = 1423). [file 1471-2458-11-579-S2.DOC]

**Table S2. Multivariate Associations [HR (95%CI)] of LS with all-cause mortality (N=2675).** Marked in bold are significant HR (p<.05)

| **Covariates** | **Crude model** | **Model CVD risk factors** | **Model health** | **Model psychological determinants** | **Model social determinants** |
| --- | --- | --- | --- | --- | --- |
| Sex: Male | **2.38 (1.78 - 3.18)** | **2.20 (1.64 - 2.95)** | **2.47 (1.84 - 3.32)** | **2.60 (1.94 - 3.49)** | **2.44 (1.82 - 3.25)** |
| Age: 60 years or older | **8.42 (6.43 - 11.03)** | **8.26 (6.20 - 11.00)** | **6.24 (4.69 - 8.30)** | **7.65 (5.81 - 10.08)** | **7.87 (5.99 - 10.34)** |
| Life satisfaction: Higher tertile LS | **0.61 (0.44 - 0.86)** | **0.68 (0.49 - 0.95)** | **0.64 (0.46 - 0.90)** | 0.77 (0.54 - 1.09) | **0.63 (0.45 - 0.88)** |
| Alcohol: ≥ 20 g/d women; ≥ 40 g/d men | - | **1.41 (1.06 - 1.86)** | - | - | - |
| Obesity: BMI ≥ 30 | - | **1.62 (1.24 - 2.12)** | - | - | - |
| Hypertension: ≥ 140/90 mm Hg | - | 1.30 (0.97 - 1.76) | - | - | - |
| Smoking: current regular smoker | - | **2.04 (1.52 - 2.75)** | - | - | - |
| Total cholesterol < 4.78 mmol/l | - | 0.73 (0.45 - 1.16) | - | - | - |
| Physical inactive: <1 h/wk | - | **1.42 (1.08 - 1.87)** | - | - | - |
| Absence of co-morbidities | - | - | **0.45 (0.34 - 0.60)** | - | - |
| Absence of medication | - | - | **0.55 (0.39 - 0.76)** | - | - |
| Low/ medium somatic complaints | - | - | - | 0.78 (0.57 - 1.05) | - |
| Depressed mood | - | - | - | 1.02 (0.75 - 1.38) | - |
| Impaired self-rated health | - | - | - | 1.23 (0.85 - 1.77) | - |
| Good health status | - | - | - | **0.55 (0.36 - 0.82)** | - |
| No disposition to irritation | - | - | - | 0.96 (0.74 - 1.24) | - |
| No anger | - | - | - | 1.08 (0.83 - 1.41) | - |
| High social network | - | - | - | **0.69 (0.53 - 0.90)** | - |
| High net income | - | - | - | - | **0.62 (0.44 - 0.87)** |

**Table S3. Multivariate Associations [HR (95%CI)] of LS with all-cause mortality in women (N=1252).** Marked in bold are significant HR (p<.05)

| **Covariates** | **Crude model** | **Model CVD risk factors** | **Model health** | **Model psychological determinants** | **Model social determinants** |
| --- | --- | --- | --- | --- | --- |
| Age: 60 years or older | **17.41 (9.57 - 31.69)** | **12.98 (6.69 - 25.21)** | **15.02 (8.12 - 27.79)** | **14.80 (7.98 - 27.43)** | **17.03 (9.33 - 31.08)** |
| Life satisfaction: Higher tertile LS | 0 93 (0.49 - 1.76) | 0.95 (0.50 - 1.80) | 0.88 (0.47 - 1.65) | 1.13 (0.58 - 2.17) | 0.93 (0.50 - 1.76) |
| Alcohol use: ≥ 20 g/d women; ≥ 40 g/d men | - | 1.45 (0.72 - 2.89) | - | - | - |
| Obesity: BMI ≥ 30 | - | 1.36 (0.79 - 2.33) | - | - | - |
| Hypertension: ≥ 140/90 mm Hg | - | 1.43 (0.76 - 2.69) | - | - | - |
| Smoking: current regular smoker | - | 1.18 (0.55 - 2.56) | - | - | - |
| Total cholesterol < 4.78 mmol/l | - | 0.47 (0.11 - 2.00) | - | - | - |
| Physical inactive: <1 h/wk | - | 1.48 (0.84 - 2.62) | - | - | - |
| Absence of co-morbidities | - | - | **0.35 (0.20 - 0.63)** | - | - |
| Absence of medication | - | - | 0.87 (0.42 - 1.82) | - | - |
| Low/ medium somatic complaints | - | - | - | 0.58 (0.31 - 1.08) | - |
| Depressed mood | - | - | - | 1.17 (0.63 - 2.16) | - |
| Impaired self-rated health | - | - | - | 0.89 (0.45 - 1.76) | - |
| Good health status | - | - | - | 0.51 (0.25 - 1.05) | - |
| No disposition to irritation | - | - | - | 0.95 (0.57 - 1.58) | - |
| No anger | - | - | - | 1.54 (0.91 -.2.59) | - |
| High social network | - | - | - | 0.86 (0.50 - 1.51) | - |
| High net income | - | - | - | - | 0.79 (0.40 - 1.56) |

**Table S4. Multivariate Associations [HR (95%CI)] of LS with all-cause mortality in men (N=1423).** Marked in bold are significant HR (p<.05)

| **Covariates** | **Crude model** | **Model CVD risk factors** | **Model health** | **Model psychological determinants** | **Model social determinants** |
| --- | --- | --- | --- | --- | --- |
| Age: 60 years or older | **6.73 (4.96 - 9.12)** | **7.07 (5.13 - 9.75)** | **4.62 (3.34 - 6.39)** | **6.37 (4.66 - 8.70)** | **6.15 (4.52 - 8.38)** |
| Life satisfaction: Higher tertile LS | **0.55 (0.37 - 0.81)** | **0.61 (0.41 - 0.91)** | **0.58 (0.39 - 0.86)** | 0.68 (0.45 - 1.02) | **0.57 (0.38 - 0.84)** |
| Alcohol use: ≥ 20 g/d women; ≥ 40 g/d men | - | **1.38 (1.01 - 1.88)** | - | - | - |
| Obesity: BMI ≥ 30 | - | **1.69 (1.24 - 2.31)** | - | - | - |
| Hypertension: ≥ 140/90 mm Hg | - | 1.21 (0.86 - 1.69) | - | - | - |
| Smoking: current regular smoker | - | **2.27 (1.63 - 3.15)** | - | - | - |
| Total cholesterol < 4.78 mmol/l | - | 0.82 (0.50 - 1.36) | - | - | - |
| Physical inactive: <1 h/wk | - | **1.37 (1.00 - 1.88)** | - | - | - |
| Absence of co-morbidities | - | - | **0.48 (0.35 - 0.67)** | - | - |
| Absence of medication | - | - | **0.46 (0.32 - 0.67)** | - | - |
| Low/ medium somatic complaints | - | - | - | 0.83 (0.59 - 1.17) | - |
| Depressed mood | - | - | - | 0.95 (0.67 - 1.35) | - |
| Impaired self-rated health | - | - | - | 1.38 (0.90 - 2.12) | - |
| Good health status | - | - | - | **0.59 (0.36 - 0.96)** | - |
| No disposition to irritation | - | - | - | 0.97 (0.72 - 1.31) | - |
| No anger | - | - | - | 0.96 (0.71 - 1.31) | - |
| High social network | - | - | - | **0.67 (0.50 - 0.90)** | - |
| High net income | - | - | - | - | **0.56 (0.38 - 0.83)** |
